# Prevention of Human Papillomavirus-Related Malignancies of the Female Genital Tract

**DOI:** 10.1155/2011/737139

**Published:** 2012-04-05

**Authors:** George Koliopoulos, Pierre Martin-Hirsch, Marc Arbyn

**Affiliations:** ^1^Department of Obstetrics and Gynecology, University Hospital of Ioannina, 45500 Ioannina, Greece; ^2^Department of Obstetrics and Gynecology, Lancashire Teaching Hospitals, Preston PR2 9HT, UK; ^3^Unit of Cancer Epidemiology, Scientific Institute of Public Health, 1050 Brussels, Belgium

Persistent infection from high-risk HPV types is necessary for the development of cervical cancer and constitutes a main cause for a significant proportion of cancers in other sites of the female anogenital tract such as the vulva, the vagina, and the anus [1]. Cervical cancer in particular being the third most common cancer in women (with approximately 530,000 cases and 275,000 deaths per year) [2] is an important global health issue warranting intensive preventive efforts [3]. Secondary prevention through cervical cytology screening has been effective in reducing the incidence and the mortality of the disease [4]. However, cervical cytology has certain limitations one of which is poor reproducibility. In the study by A. C. Barcelos et al. titled “*Atypical squamous cells of undetermined significance: bethesda classification and association with human papillomavirus*” which appears in this special issue, observers would agree in the diagnosis of ASCUS only in two out of three cases.

Secondary screening efforts focus on the detection and subsequent treatment of cervical intraepithelial neoplasia (CIN). However, the sensitivity of cytology in the detection of CIN has been questioned [5]. Obviously failure of this mechanism might result in the development of cervical cancer. HPV DNA testing emerged as a way to improve the deficiencies of cytology [6]. The study by Barcelos et al. [7] confirmed that HPV DNA testing with Hybrid Capture II (HCII) can be applied in the triage of ASCUS cytology. Interestingly the HCII positivity rate in this cohort of ASCUS smears (22%) was lower than in the ALTS (55%) [8].

HCII, however, does not allow HPV typing. Certain PCR-based tests allow the identification of the specific genotypes responsible for the infection, which could have future clinical applications. One of the first steps in PCR is DNA extraction. The study by Chranioti et al. [9] compared the accuracy of the method after manual and after automated DNA extraction and found better results with the former.

Cell cycle biomarkers such as E6&7 mRNA testing and P16INK4a are further tools that could be used in the prevention of cervical cancer [10]. However, biomarkers could also have prognostic value and influence management of cervical (pre-)cancer. In the study by F. M. Abu Backer et al. titled “*Clinicopathological comparison of adenocarcinoma of cervix and endometrium using cell cycle markers: P16ink4a, P21waf1, and p27Kip1 on 132 Cancers*” the expression of two less studied proteins, p21WAF1 and p27Kip1 was significantly associated with lymph node invasion in cervical adenocarcinoma.

Most HPV infections are transient without clinical significance. However, in a minority of women HPV will persist and will initiate the neoplastic process. Epigenetic event such as gene methylation might play a role in the selection of the women who will progress to cancer. The study by A. Spathis et al. titled “*Promoter methylation of p*16^*INK*4*A*^ *, hMLH1, and MGMT in liquid-based cervical cytology samples compared with clinicopathological findings and HPV presence*” examined the methylation-dependent inactivation of tumor suppressor genes connected with cell cycle regulation as p16INK4A and DNA repair mechanisms as human MutL Homolog 1 (hMLH1) and O6-methylguanine DNA methyl transferase (MGMT). Even though the accuracy of methylation was found lower than traditional tests, epigenetic effects are areas of promising future research.

The deficiencies of secondary prevention, such as the need for sufficient infrastructure, which is absent in developing countries, the false negative results, the adverse effects of overtreatment on subsequent pregnancy outcome [11, 12], as well as the psychological morbidity resulting from HPV infection [13] have left plenty of room for improvement. With the introduction of HPV vaccines a combination of secondary with primary prevention is now possible [14, 15]. The article by K. Natunen et al. titled “*Aspects of prophylactic vaccination against cervical cancer and other human papillomavirus-related cancers in developing countries*” outlines certain issues that arise with respect to application of the vaccine in the developing countries where the need for cervical cancer prevention is the greatest. They suggest that given the low coverage rates, vaccination of the males might have to be considered in order to produce herd immunity. They also argue that strategies for vaccine implementation would have to be school based with community outreach activities in regions where school attendance is low. 

Progress in the elucidation of the mechanisms of carcinogenesis has led to the development of exciting technologies that can be used for lower anogenital tract cancer prevention. The main challenge we face now is to find the proper way to use all these discoveries for the benefit of women's health. 

**George Koliopoulos** **George Koliopoulos** **Pierre Martin-Hirsch** **Pierre Martin-Hirsch** **Marc Arbyn** **Marc Arbyn**
